# Efficacy of fumigation with traditional Chinese medicine combined with Acanthopanax Senticosus injection in patients with sleep disorders

**DOI:** 10.12669/pjms.41.11.12571

**Published:** 2025-11

**Authors:** Yanying Hu, Xiaoxian Guo, Yan Gao, Wenli Li, Jing Kang

**Affiliations:** 1 Yanying Hu, Department of Neurology, Yan’an Hospital of Traditional Chinese Medicine, Yan’an Branch of Peking University Third Hospital, Yan’an, Shaanxi Province 716000, P.R. China; 2 Xiaoxian Guo, Department of Neurology, Yan’an Hospital of Traditional Chinese Medicine, Yan’an Branch of Peking University Third Hospital, Yan’an, Shaanxi Province 716000, P.R. China; 3 Yan Gao, Department of Neurology, Yan’an Hospital of Traditional Chinese Medicine, Yan’an Branch of Peking University Third Hospital, Yan’an, Shaanxi Province 716000, P.R. China; 4 Wenli Li, Department of Neurology, Yan’an Hospital of Traditional Chinese Medicine, Yan’an Branch of Peking University Third Hospital, Yan’an, Shaanxi Province 716000, P.R. China; 5 Jing Kang, Department of Neurology, Yan’an Hospital of Traditional Chinese Medicine, Yan’an Branch of Peking University Third Hospital, Yan’an, Shaanxi Province 716000, P.R. China

**Keywords:** Acanthopanax senticosus injection, Fumigation and washing of traditional Chinese medicine, Sleep disorders

## Abstract

**Objective:**

To evaluate the efficacy of traditional Chinese medicine (TCM) fumigation and washing combined with Acanthopanax senticosus injection in patients with sleep disorders.

**Methodology:**

The clinical data total of 106 patients with sleep disorders treated at Yan’an Traditional Chinese Medicine Hospital from January 2023 to January 2025 were retrospectively analyzed. Patients were divided into two groups: the control group (n=51) received routine care with dexzopiclone; the study group (n=55) received additional TCM fumigation and Acanthopanax senticosus injection. Sleep quality was assessed using the Pittsburgh Sleep Quality Index (PSQI), sleep structure was monitored via polysomnography (PSG), and quality of life was evaluated using the GQOLI-74 scale. Safety indicators and adverse events were also recorded.

**Results:**

After the intervention, the study group showed significantly greater improvements in PSQI scores compared to the control group (P<0.001). PSG parameters indicated fewer nocturnal arousals, shorter arousal time and sleep latency, and higher sleep efficiency in the study group (P<0.05). Quality of life scores across physical, psychological, social, and mental domains also improved significantly more in the study group. No adverse events were reported during the study.

**Conclusions:**

TCM fumigation and washing combined with Acanthopanax senticosus injection may serve as an effective, well-tolerated, and non-invasive therapy for improving sleep quality and life quality in patients with sleep disorders. The therapy may be particularly beneficial for individuals with poor tolerance to hypnotic medications or stress-related insomnia. Further prospective randomized trials with longer follow-up are warranted to confirm long-term efficacy.

## INTRODUCTION

Sleep is a key physiological process that regulates the physiological function and metabolic activity of the human body at the molecular, cellular and systemic levels.^1^,^2^ In recent years, due to the change of social environment and lifestyle and the increase in mental stress, the prevalence of sleep disorders has been on the rise and has become a public health problem.^3^,^4^ Long-term sleep disorders not only interfere with the normal rhythm of the neuroendocrine system, resulting in hormone imbalance, but also impair neurocognitive function, promote abnormal blood pressure fluctuations and heart rate variability and increase the risk of cardiovascular and cerebrovascular events.^5^,^6^

Currently, the treatment of sleep disorders mainly involves pharmaceuticals and psychological intervention. Although drug treatment can improve sleep symptoms to a certain extent, long-term use of benzodiazepines, non-benzodiazepines and other drugs may lead to drug tolerance and adverse reactions, which seriously affect the quality of life and treatment compliance of patients.^7^,^8^ Although cognitive behavioral therapy and other psychological intervention measures adjust patients’ sleep cognition and behavior patterns from the psychological level, they require long-term professional guidance, a prolonged treatment cycle and patients’ cooperation and self-regulation ability.^9^,^10^

Traditional medicine has rich clinical practice experience in the field of prevention and treatment of sleep disorders. Traditional Chinese medicine (TCM) fumigation and washing is an external treatment method based on the theory of meridians and skin penetration. The warm temperature of the medicine ensures that the effective ingredients permeate through the skin and follow the meridian through which the QI and blood flow to the disease center.^11^ Acanthopanax senticosus injection, rich in various active ingredients, is extensively used in TCM due to its tonifying action on the kidney and calming effect, benefiting QI and invigorating the spleen.^12^ Modern pharmacological research has demonstrated the benefits of Acanthopanax senticosus in improving sleep due to its ability to regulate the level of neurotransmitters, alleviate oxidative stress and improve the neuroendocrine function.^13^ Recent evidence suggests that the combination of TCM fumigation and Acanthopanax Senticosus injection may act synergistically through both peripheral and central regulatory mechanisms.^13^-^15^ Acanthopanax Senticosus has been shown to modulate the hypothalamic–pituitary–adrenal (HPA) axis, rebalance cortisol secretion, and influence circadian rhythms by altering the expression of monoamine neurotransmitters such as serotonin (5-HT) and dopamine (DA).^14^ Meanwhile, TCM fumigation, via transdermal absorption and meridian conduction, is thought to improve autonomic nervous system function and peripheral microcirculation.^15^ Together, these interventions may reinforce sleep initiation and maintenance by modulating sleep architecture, reducing arousal events, and promoting sleep continuity. This study aimed to investigate the application effect of the combination of Chinese herbal fumigation and Acanthopanax senticosus injection in patients with sleep disorders.

## METHODOLOGY

The clinical data of 106 patients with sleep disorders admitted to Yan’an Traditional Chinese Medicine Hospital between January 2023 to January 2025 were retrospectively selected. Fifty one patients received the routine treatment and were set as the control group, while 55 patients who were treated with the Chinese medicine (Wen Yang Huo Xue Anshen Fang) fumigation and Acanthopanax senticosus injection comprised the study group. All patients included in the analysis met the predefined inclusion and exclusion criteria and completed the full course of intervention and assessment. As the study was based on retrospective data retrieved from the hospital’s electronic medical record (EMR) system, no patients were lost to follow-up or dropped out. Cases with incomplete medical records were excluded during the initial screening phase and thus are not part of the final dataset.

### Ethical approval:

The ethics committee of our hospital approved the study, No. 202504-18, Date: April 18^th^, 2025. This study was conducted in full accordance with the Declaration of Helsinki. As a retrospective study, only previously recorded clinical data were analyzed. All patient records were de-identified and anonymized before data extraction to ensure confidentiality. Data processing and statistical analysis were performed within the secure hospital intranet.

### Inclusion criteria:

- Meets sleep disorder diagnosis criteria.^16^
- Age: 18~70.
- Course of disease: ≧ months.
- Normal liver and kidney function.
- Signed informed consent.

### Exclusion criteria:

- Malignant tumor.
- Failure of liver and kidney function.
- Diagnosis of schizophrenia and mania.
- Patients with a history of alcohol or drug abuse or other addictions.
- Patients with severe dermatosis (such as large area eczema, skin ulcer, allergic dermatitis, etc.).
- Allergic constitution.
- Participate in other sleep-related researchers at the same time.

### Routine treatment (control group):

routine sleep hygiene education for patients; guiding the patients to establish regular work and rest time, try to go to bed and get up at the same time every day, ensure the bedroom is quiet, comfortable, dark and cool and avoid using electronic equipment at night to affect sleep behavior. Patients were administered Dexzopiclone tablets 3-mg/times orally once a night. Total length of the treatment is two weeks.

### Combined treatment (study group):

In addition to the routine treatment, patients received Chinese medicine (Wen Yang Huo Xue Anshen Fang) fumigation and washing combined with Acanthopanax senticosus injection. Chinese medicine fumigation and washing: 9g of Coptis chinensis, 9g of Cortex Cinnamomi, 15g of Albizia bark, 21g of magnet, 9g of Artemisia argyi, 6-g of safflower, 6-g of Pericarpium Zanthoxyli, 9g of Fructus Evodiae and 15-g of Poria cum Radix Pini were grinded, put into cloth bag, soaked in 2000 ml water for 30 minutes and boiled for 30 minutes. When the medicinal liquid was cooled to 40-45°C, patients were instructed to soak their feet in the medicinal liquid up to the ankle joint. Each fumigation and washing was performed for 20- 30 minutes, one hour before bedtime every night. Additionally, patients were administered Acanthopanax senticosus injection at a dose of 60 ml once daily for 10 consecutive days. This dosage was selected based on official prescribing guidelines approved by the National Medical Products Administration (NMPA) of China, which recommend a therapeutic range of 30–60 ml/day for neurological fatigue, neurasthenia, and related conditions. Clinical protocols in tertiary hospitals also commonly adopt 60 ml/day as the upper limit of safe and effective short-term use, particularly in 1–2 weeks treatment cycles. This dose has been shown to exert neuroprotective and anti-fatigue effects via regulation of the HPA axis and monoamine neurotransmitter levels, which may contribute to sleep regulation.^17^ During the entire treatment period, a safety monitoring protocol was implemented to observe any adverse reactions, including but not limited to skin irritation, dizziness, or allergic responses. No adverse events were observed in any participant.

### Collected observation indexes:

- Before and after intervention, the sleep quality of the two groups was measured by the Pittsburgh Sleep Quality Index (PSQI), which included sleep time, sleep quality and seven other dimensions. The score range of each dimension was 0~3. The lower score indicated better sleep quality.
- The sleep structure was assessed in both groups before and after the intervention using standard polysomnography (PSG) conducted in the hospital’s dedicated sleep monitoring room. The environment was temperature-controlled, quiet, and shielded from external light or noise. Each participant underwent a single overnight PSG session, starting at 10:00 p.m. and ending at 6:00 a.m., with continuous monitoring for approximately 8 hours. Parameters including the number of arousals, total arousal time, sleep efficiency, and sleep latency were recorded. All PSG recordings were independently scored by two certified sleep technicians who were blinded to the treatment allocation to minimize assessment bias.
- The quality of life before and after the intervention was assessed using the General Quality of Life Inventory-74 (GQOLI-74), a culturally adapted instrument widely used in Chinese clinical settings, particularly in studies involving chronic diseases and Traditional Chinese Medicine (TCM) interventions. This tool evaluates four key dimensions: physiological function, psychological function, social function, and mental well-being. GQOLI-74 was selected over internationally standardized instruments such as SF-36 or WHOQOL due to its greater contextual relevance, language compatibility, and familiarity among Chinese patients and clinicians. Each subscale is scored from 0 to 100, with higher scores indicating better quality of life.
- Safety indicators were monitored throughout the intervention. Patients were closely observed for any signs of skin irritation, dizziness, allergic reactions, or injection-related discomfort. No adverse events were reported during the entire treatment and observation period.

### Statistical analysis:

SPSS 25.0 was used for the analysis. The measurement data was presented as (±s) and a t-test was used. Counting data was expressed as frequency and composition ratio (%) and <unk> two test was used. P<0.05 indicated a statistically significant difference.

## RESULTS

There was no statistical significance (P>0.05) between the two groups in general characteristics such as gender, age, course of disease, basic diseases, etc. (Table-I).

**Table-I:** Comparison of general data between two groups.

Item	Study Group(n=55)	Contrast Group(n=51)	t/χ^2^Value	P-Value
***Sex[n(%)]***				
Male	29(52.73)	30(58.82)	0.399	0.528
Female	26(47.27)	21(41.18)		
Age(χ̅±S, year)	59.78±12.38	61.07±10.96	0.566	0.572
Course of diseases(χ̅±S, month)	10.24±4.19	9.77±5.02	0.525	0.601
***Hypertensive[n(%)]***				
With	24(43.64)	21(41.18)	0.066	0.798
Without	31(56.36)	30(58.82)		
***Diabetes[n(%)]***				
With	11(20.00)	12(23.53)	0.194	0.660
Without	44(80.00)	39(76.47)		
***Coronary disease [n(%)]***				
With	15(27.27)	10(19.61)	0.863	0.353
Without	40(72.73)	41(80.39)		

### Sleep quality:

As shown in Table-II, there was no significant difference in daytime dysfunction, dosage of hypnotics, sleep disorder, sleep quality, sleep time and sleep quality scores between the two groups before intervention (P>0.05). After intervention, the two groups’ daytime dysfunction, dosage of hypnotics, sleep disorder, sleep quality, sleep time and sleep quality score were lower than before intervention and considerably lower in the study group than in the control group (P<0.05).

**Table-II:** Comparison of sleep quality between two groups(*χ̅*±*S*, min).

Item	Group	Count	Daytime dysfunction	Dosage of hypnotics		Sleep disorder	Sleep Efficiency	Sleep time	Time to sleep	Sleep Quality
Before intervention	Study group	55	1.74±0.54	1.22±0.18	1.37±0.26		1.96±0.28	1.88±0.32	1.77±0.30	2.16±0.53
	Comparison group	51	1.76±0.51	1.24±0.15	1.39±0.24		1.94±0.31	1.90±0.30	1.80±0.26	2.19±0.50
	t Value		0.196	0.619	0.411		0.349	0.331	0.548	0.299
	P Value		0.845	0.537	0.682		0.728	0.741	0.585	0.765
After intervention	Study group	55	0.89±0.24	0.63±0.12	0.77±0.14		1.02±0.19	0.92±0.20	0.91±0.17	1.07±0.24
	Comparison group	51	1.27±0.30	0.76±0.14	0.95±0.23		1.17±0.22	1.15±0.24	1.13±0.20	1.41±0.31
	T Value		7.226	5.144	4.907		3.765	5.375	6.116	6.340
	P Value		<0.001	<0.001	<0.001		<0.001	<0.001	<0.001	<0.001

### Sleep structure:

There was no significant difference in the number of arousals, time of arousal, sleep quality, and sleep incubation period between the two groups before intervention (P>0.05). After intervention, the number of arousals, time of arousal and sleep incubation period in the two groups decreased and the sleep quality improved significantly after the intervention. The number of arousals, time of arousal and sleep incubation period in the study group were shorter than those in the control group and the sleep quality, was significantly higher (P<0.05) Table-III.

**Table-III:** Comparison of sleep structure between two groups(*χ̅*±*S*).

Item	Group	Count	Number of arousal (time)	Time of arousal (min)	Sleep quality (%)	Sleep latency (min)
Before intervention	Study group	55	5.94±1.19	118.64±13.57	79.80±6.19	61.66±16.51
	Comparison group	51	6.06±1.28	121.02±10.61	80.22±5.81	62.37±18.62
	t Value		0.500	1.001	0.359	0.208
	P Value		0.618	0.319	0.720	0.836
After intervention	Study group	55	2.18±0.59	68.36±6.12	88.08±7.26	24.42±3.37
	Comparison group	51	3.32±0.65	75.09±8.60	84.05±6.71	26.27±4.08
	t Value		9.465	4.668	2.961	2.553
	P Value		<0.001	<0.001	0.004	0.012

### Quality of life:

As shown in Table-IV, there was no significant difference in the scores of mental state, psychological function, social function and physical function between the two groups before intervention (P>0.05). In contrast, post-intervention QOL scores of the two groups became considerably higher compared to pre-intervention values and were significantly better in the study group (P<0.05) Table-IV.

**Table-IV:** Comparison of QOL between two groups (*χ̅*±*S*, min).

Item	Group	Count	Mental state	Psychological function	Social function	Physical function
Before intervention	Study group	55	69.02±6.51	66.42±7.79	65.72±5.44	73.11±5.65
	Comparison group	51	68.80±7.42	67.11±6.54	64.68±6.25	71.69±6.47
	t Value		0.163	0.492	0.916	1.206
	P Value		0.871	0.624	0.362	0.231
After intervention	Study group	55	88.24±4.80	90.02±6.16	89.36±4.26	91.71±6.77
	Comparison group	51	84.14±5.60	85.45±7.21	86.01±5.02	85.82±7.14
	t Value		4.056	3.516	3.713	4.359
	P Value		<0.001	0.001	<0.001	<0.001

## DISCUSSION

This study explored the quality of combining TCM fumigation and washing with Acanthopanax senticosus injection therapy in treating patients with sleep disorders. The observed improvements in sleep quality and structure may result from a synergistic interaction between central and peripheral mechanisms activated by the combined intervention.^16^ Acanthopanax Senticosus has been reported to regulate the hypothalamic–pituitary–adrenal (HPA) axis, rebalance cortisol rhythms, and modulate monoamine neurotransmitters such as serotonin and dopamine, which are essential in sleep-wake regulation.^17^ Meanwhile, TCM fumigation via transdermal absorption may stimulate cutaneous nerve endings and activate the “skin–brain axis,” a pathway increasingly recognized in neuroimmunology, potentially modulating autonomic tone and emotional reactivity.^18^ Importantly, no adverse events were observed throughout the study, and none of the participants reported skin-related reactions, dizziness, or injection-site discomfort. This suggests that the combined intervention has a favorable short-term safety profile. Nevertheless, systematic safety monitoring remains essential, especially in larger populations and longer-term applications.

The study showed that the scores of each dimension of PSQIs in the two groups were significantly decreased after intervention and were considerably lower in patients who received the combined treatment regimen. Together, these mechanisms may reduce arousal thresholds, enhance parasympathetic dominance, and stabilize sleep architecture—particularly beneficial for patients with hyperarousal-based insomnia or comorbid anxiety.^19^ In this context, the treatment protocol may represent a non-invasive, well-tolerated alternative to sedative-hypnotics, especially for populations who are drug-intolerant, elderly, or suffering from stress-related sleep disturbance. A study by Feng G et al.^17^ found that Tui Na, a form of massage therapy within TCM, is efficacious in improving daytime dysfunction and reducing the use of hypnotic drugs. Many medicinal components of the TCM play a role in tranquilizing the mind and helping sleep.^18^

Anyang Huoxue Anshen Fumigation and Washing Prescription is traditionally described as clearing heat and drying dampness (i.e., anti-inflammatory and fluid balance regulation), eliminating fire and detoxifying (removing pro-inflammatory stimuli), and alleviating insomnia caused by internal heat disturbing the mind (neuroinflammation-induced arousal); Cinnamon is believed in TCM to replenish fire to support yang (enhancing metabolic and hormonal activity), balance yin and yang (neuroendocrine homeostasis), and improve dysfunctions related to the heart and kidney systems (cardiac-autonomic regulation and renal neuroaxis); Albizia is used to relieve depression and tranquilize the mind (sedative and anxiolytic effects on the central nervous system), thereby alleviating insomnia linked to emotional dysregulation such as anxiety or depressive symptoms. Magnetite is traditionally used to calm the mind and promote sleep (modulating CNS excitability), while Artemisiae Argyi is believed to warm the meridians and dispel cold (improving peripheral circulation and autonomic balance), thus facilitating restful sleep; It can improve blood circulation and provide good physical condition for sleep; Prickly ash warm middle analgesic, insecticidal antipruritic, can adjust the body’s Qi and blood movement, relieve physical discomfort; Fructus Evodiae disperses cold and stops nausea and vomiting, which helps to regulate the function of viscera; Poria cum Radix Pini calms the heart and tranquilizes the mind and has a good effect on palpitations and insomnia. Fumigation and washing prescription drugs penetrate the skin, act on the meridian, Qi and blood and regulate the balance between internal organs and yin and yang. Acanthopanax senticosus injection was shown to regulate the function of the human body as a whole, enhance the adaptability to stress and further improve the sleep quality.^19^

In addition, the prevalence and complexity of sleep disorders are often compounded by comorbid psychiatric conditions, including anxiety and bipolar spectrum disorders. Previous studies have shown that specific populations, such as patients with bipolar disorder, are at increased risk for sleep-related complications including obstructive sleep apnea (OSA), which may further disrupt sleep architecture and reduce treatment responsiveness. These findings underscore the importance of integrated management strategies and individualized intervention models for patients with complex sleep comorbidities.^20^

Moreover, the results align with the emerging global interest in integrative and functional approaches to sleep medicine. As a low-risk, multi-target intervention, the combined use of TCM fumigation and Acanthopanax Senticosus injection may provide meaningful benefits when incorporated into personalized care plans.^21^ Future studies may explore its role as an adjunct to cognitive behavioral therapy for insomnia (CBT-I) or as a primary modality in resource-limited settings. The sleep quality was significantly improved in both groups and the improvement degree of the above indexes in the study group was better than that in the control group (P<0.05). Hu H et al.^21^ found that acupuncture and moxibustion effectively improved sleep structure. However, their study reported a significant individual difference and a short duration of effect. Modern pharmacology studies show that the drug components in the fumigation and washing prescription can act on the nervous system, regulate the secretion and release of neurotransmitters and further optimize the sleep structure.^22^-^24^ Since Acanthopanax senticosus injection can regulate the excitement and inhibition of the central nervous system, the synergistic effect of the combined treatment regimen may cause the patient to fall asleep faster and sleep more stably, with fewer arousal times.

According to the QOL assessment, the scores of mental status, psychological function, social function and physical function were improved in both groups after the intervention and the improvement was greater in the study group (P<0.05). A study by Espie CA et al.^25^ showed that while the digital cognitive behavioral therapy alone has a certain effect on improving psychological function, it has a limited effect on improving physical function and social function of patients with sleep disorders. Sleep disorders are often accompanied by problems such as anxiety and depression, which seriously affect the social communication and daily life of patients.

### Strength of the study:

First, it is among the first clinical investigations to evaluate the combined use of traditional Chinese medicine (TCM) fumigation and Acanthopanax senticosus injection in managing sleep disorders, offering novel insights into integrative therapeutic approaches. Second, the inclusion of objective sleep structure assessment via polysomnography (PSG) enhances the methodological rigor and adds credibility to the findings. Third, the study benefits from a relatively large sample size and a controlled comparison design, which improves internal validity. However, further research is warranted to build upon these findings. Future studies should employ prospective randomized controlled trial (RCT) designs to minimize selection and confounding biases. Long-term efficacy and relapse risk should be evaluated through extended follow-up periods beyond the current two-week window. Moreover, the underlying biological mechanisms—particularly the effects on neuroendocrine regulation and circadian rhythm—should be explored using biomarker assays or neuroimaging techniques. These efforts will help confirm the observed effects and support broader clinical adoption of this non-pharmacological intervention.

This study showed that combining TCM fumigation and washing with Acanthopanax senticosus injection has effectively alleviated these accompanying symptoms while improving sleep. Good sleep promotes brain function recovery, alleviates psychological pressure and strengthens patients’ social adaptability and self-care ability. The results of this study further confirm that combination therapy more effectively and comprehensively improves patients’ quality of life compared with an approach that relies only on psychological guidance, but has a limited impact on the physiological function.

### Limitations:

First, as a retrospective, non-randomized, single-center analysis, the study is susceptible to selection bias and unmeasured confounding variables, such as baseline sleep severity, psychological status, and patient adherence. Although strict inclusion and exclusion criteria were applied, and baseline characteristics between groups were balanced, causality cannot be definitively established due to the observational nature of the study. Future multicenter prospective randomized controlled trials (RCTs) are needed to validate these findings and strengthen the level of evidence. Second, the absence of a placebo or sham-fumigation control group may have introduced expectancy bias, particularly affecting subjective outcomes such as the Pittsburgh Sleep Quality Index (PSQI). Traditional Chinese Medicine (TCM) fumigation involves multisensory components—heat, herbal aroma, and therapeutic ritual—which may contribute to perceived improvement via non-specific mechanisms like sensory comfort and emotional reassurance. While the inclusion of objective polysomnography (PSG) parameters partially mitigates this concern, we recommend that future studies incorporate sham interventions (e.g., non-active herbs, thermal steam only) to better isolate the specific therapeutic effects. Third, the intervention and follow-up period were limited to two weeks, which may be sufficient for short-term symptom relief but inadequate to assess long-term efficacy or relapse risk. Insomnia is a chronic and relapsing disorder; thus, extended follow-up periods (e.g., 3–6 months) are necessary to evaluate sustained treatment effects and inform maintenance strategies. Lastly, the relatively small sample size and lack of in-depth mechanistic exploration limit the generalizability of the findings. Future studies should involve larger cohorts and explore the underlying biological pathways—such as neuroendocrine regulation and circadian rhythm modulation—to optimize the therapeutic approach.

## CONCLUSION

The combination of traditional Chinese medicine fumigation and washing with Acanthopanax senticosus injection appears to be an effective, non-invasive therapeutic option for improving sleep structure, quality, and overall quality of life in patients with sleep disorders. This approach may be particularly beneficial for individuals who are intolerant to conventional hypnotic medications or who suffer from chronic insomnia associated with psychological stress, anxiety, or autonomic nervous system dysregulation. Given its favorable safety profile and ease of administration, this therapy could be integrated into clinical practice in traditional Chinese medicine rehabilitation units, integrative sleep clinics, or community-level outpatient settings. Further large-scale, prospective studies are recommended to validate its long-term efficacy and optimize treatment protocols across diverse patient populations.

### Authors’ contributions:

**YH:** Study design, literature search and manuscript writing.

**XG, YG, WL and JK:** Data collection, data analysis and interpretation. Critical Review.

**YH:** Manuscript revision and validation and is responsible for the integrity of the study.

All authors have read and approved the final manuscript.

## References

[1] Jia X, Song Y, Li Z, Yang N, Liu T, Han D (2024). Melatonin regulates the circadian rhythm to ameliorate postoperative sleep disorder and neurobehavioral abnormalities in aged mice. CNS Neurosci Ther..

[2] Ahlberg R, Garcia-Argibay M, Taylor M, Lichtenstein P, D'Onofrio BM, Butwicka A (2023). Prevalence of sleep disorder diagnoses and sleep medication prescriptions in individuals with ADHD across the lifespan:a Swedish nationwide register-based study. BMJ Ment Health..

[3] Lugo J, Fadeuilhe C, Gisbert L, Setien I, Delgado M, Corrales M (2020). Sleep in adults with autism spectrum disorder and attention deficit/hyperactivity disorder:A systematic review and meta-analysis. Eur Neuropsychopharmacol J Eur Coll Neuropsychopharmacol..

[4] Wang Y, Fan H, Ren Z, Liu X, Niu X (2023). Sleep disorder, Mediterranean diet and all-cause and cause-specific mortality:a prospective cohort study. BMC Public Health..

[5] Szûcs A, Mutti C, Papp A, Halász P, Parrino L (2022). REM sleep, REM parasomnias, REM sleep behaviour disorder. Ideggyogyaszati Szle..

[6] Fabbri M, Beracci A, Martoni M, Meneo D, Tonetti L, Natale V (2021). Measuring Subjective Sleep Quality:A Review. Int J Environ Res Public Health..

[7] Weber FC, Wetter TC (2022). The Many Faces of Sleep Disorders in Post-Traumatic Stress Disorder:An Update on Clinical Features and Treatment. Neuropsychobiology..

[8] Sun Y, Tisdale RK, Kilduff TS (2021). Hypocretin/Orexin Receptor Pharmacology and Sleep Phases. Front Neurol Neurosci..

[9] Sun SY, Chen GH (2022). Treatment of Circadian Rhythm Sleep-Wake Disorders. Curr Neuropharmacol..

[10] Langstengel J, Yaggi HK (2022). Sleep Deficiency and Opioid Use Disorder:Trajectory, Mechanisms and Interventions. Clin Chest Med.

[11] Xu K, Zhu W, Shi H, Wang L, Wang X, Yuan Y (2019). Visual Analysis of the Research Status and Trend of Insomnia Treated by Traditional Chinese Medicine in Recent Twenty Years Based on CNKI. Chin J Inf Tradit Chin Med.

[12] Huang L, Zhao H, Huang B, Zheng C, Peng W, Qin L (2011). Acanthopanax senticosus:review of botany, chemistry and pharmacology. Pharm..

[13] Guan L (2017). Clinical observation on treatment of insomnia with acanthopanax senticosus fluid extract. J Clin Tradit Chin Med..

[14] Gu X, Zhang G, Wang Q, Song J, Li Y, Xia C (2022). Integrated network pharmacology and hepatic metabolomics to reveal the mechanism of Acanthopanax senticosus against major depressive disorder. Front Cell Dev Biol..

[15] Zhang Z, Wu Y, Shi D, Jiang C, Cao H, Jiang F (2024). Acanthopanax senticosus improves cognitive impairment in Alzheimer's disease by promoting the phosphorylation of the MAPK signaling pathway. Front Immunol..

[16] Smith MT, McCrae CS, Cheung J, Martin JL, Harrod CG, Heald JL (2018). Use of Actigraphy for the Evaluation of Sleep Disorders and Circadian Rhythm Sleep-Wake Disorders:An American Academy of Sleep Medicine Clinical Practice Guideline. J Clin Sleep Med.

[17] Feng G, Han M, Li X, Geng L, Miao Y (2019). Clinical effectiveness of Tui Na for insomnia compared with estazolam:A systematic review and meta-analysis of randomized controlled trials. Complement Ther Med..

[18] Zhang Q, Zhang X (2023). Study on Medication Law of Insomnia Doctors in Ming and Qing Dynasties Based on Data Mining. World J Sleep Med.

[19] Lau KM, Yue GGL, Chan YY, Kwok HF, Gao S, Wong CW (2019). A review on the immunomodulatory activity of Acanthopanax senticosus and its active components. Chin Med.

[20] Zhu F, Cui C, Gu K (2025). Prevalence and predictors of obstructive sleep apnea in patients with bipolar disorder:A systematic review and meta-analysis. Pak J Med Sci..

[21] Hu H, Li Z, Cheng Y, Gao H (2022). The Efficacy and Safety of Acupuncture for Depression-Related Insomnia:Protocol for a Systematic Review and Meta-Analysis. J Pain Res..

[22] Cui H, Zhao Y, Ju C, Hao J (2021). The effectiveness of traditional Chinese medicine fumigation and washing nursing care after arthroscopic debridement of Knee Osteoarthritis:A protocol for systematic review and meta-analysis. Medicine (Baltimore)..

[23] Wang XL, Zhu XP, Ji DX, Wang J, Zhai RH, Li P (2018). Beneficial effect of traditional Chinese medicine fumigation “Bone-healing Powder”in postoperative pain and recovery of neurological function of traumatic thoracolumbar spine fractures:A case-control study. Medicine (Baltimore)..

[24] Zhang L, Tian X, Ma Y, Jin YH, Meng FJ (2015). Efficacy of combining traditional Chinese medicine fumigation with Western medicine for diabetic peripheral neuropathy:A systematic review and meta-analysis. Int J Nurs Sci..

[25] Espie CA, Emsley R, Kyle SD, Gordon C, Drake CL, Siriwardena AN (2019). Effect of Digital Cognitive Behavioral Therapy for Insomnia on Health, Psychological Well-being and Sleep-Related Quality of Life:A Randomized Clinical Trial. JAMA Psychiatry..

